# Metabolic syndrome severity and all-cause mortality in the CLHLS biomarker subsample of older Chinese adults

**DOI:** 10.3389/fpubh.2026.1832339

**Published:** 2026-05-29

**Authors:** Chongyu Ding, Darong Hao, Jianghua Huo, Yaqian Xu, Hui Zhang, Yulu Gong, Xuetong Zhao, Zhaojun Wang, Xiangwei Li

**Affiliations:** 1 School of Global Health, Chinese Centre for Tropical Diseases Research, Shanghai Jiao Tong University School of Medicine, Shanghai, China; 2 School of Public Health, Shanghai Jiao Tong University School of Medicine, Shanghai, China; 3 School of Public Health and Management, Jiangsu Medical College, Yancheng, Jiangsu, China; 4 Hainan International Medical Center, Shanghai Jiao Tong University School of Medicine, Qionghai, Hainan, China

**Keywords:** all-cause mortality, Chinese older population, CLHLS, metabolic syndrome, MetS Score

## Abstract

**Background:**

Metabolic syndrome (MetS) is a cluster of risk factors that increases cardiometabolic disease and mortality. A continuous MetS Score has been developed to quantify MetS severity, but its association with all-cause mortality in older Chinese adults remains unclear.

**Methods:**

We analyzed 2,443 participants from the Chinese Longitudinal Healthy Longevity Survey (CLHLS, 2008–2018 and 2011–2018). MetS Score was derived by confirmatory factor analysis using triglycerides, HDL-C, fasting glucose, systolic blood pressure, and BMI. Participants were categorized into quartiles (Q1-Q4). Cox proportional hazards models estimated hazard ratios (HRs) and 95% confidence intervals (CIs) for all-cause mortality. Subgroup analyses examined heterogeneity by age, sex, residence, and other covariates, and restricted cubic splines explored dose–response relationships.

**Results:**

During 10,356 person-years of follow-up, 1,412 deaths occurred (136.3/1,000 person-years). Each 1-unit increase in MetS Score was associated with a 13.9% lower mortality risk (HR = 0.861, 95% CI: 0.787–0.942). The protective effect was concentrated in Q4 vs. Q1 (HR = 0.792, 95% CI: 0.669–0.938). Subgroup analyses showed stronger associations in those aged ≥80 years (HR = 0.785, 95% CI: 0.660–0.935), women (HR = 0.758, 95% CI: 0.617–0.931) and rural residents (HR = 0.817, 95% CI: 0.618–0.980). Restricted cubic splines confirmed a linear inverse association overall and among the ≥80 years group.

**Conclusion:**

MetS Score, reflecting MetS severity, was inversely associated with all-cause mortality in older Chinese adults, particularly among those aged ≥80 years. These findings suggest that higher MetS Score may paradoxically confer survival benefits in the older adults, warranting further mechanistic studies.

## Introduction

Metabolic syndrome (MetS), defined as a cluster of cardiometabolic abnormalities including central obesity, insulin resistance, hypertension, and dyslipidemia, has become a major global public health concern (1, 2). Substantial evidence indicates that both MetS and its individual components independently increase the risk of cardiovascular diseases (CVDs) and related adverse outcomes (3–5). A recent study demonstrated that the global prevalence of MetS increased from 11.9% in 2000 to 28.4% in 2023, affecting approximately 1.54 billion adults worldwide (6). In China Mainland, the prevalence of MetS also has increased strikingly to 24.5% in two decades (7). This striking increasing trend is mainly attributed to population aging, urbanization, and lifestyle changes associated with rapid economic development (6, 7). These findings highlight the urgent need for specific assessment strategies for metabolic syndrome in aging populations.

Conventional definitions, such as the NCEP ATP III criteria (8), classify MetS when three or more abnormalities are present. While clinically straightforward, this approach cannot capture the severity of metabolic dysfunction. To address this limitation, researchers have proposed continuous severity scores (“MetS Score”) (9–11), derived through confirmatory factor analysis of triglycerides, HDL-C, fasting glucose, blood pressure, and adiposity indices. These scores provide a graded measure of risk and outperform categorical definitions in predicting diabetes, CVD events, and related biomarkers (12–14). Notably, a recent Chinese study developed an age-, sex-, and ethnicity-specific MetS Score, which showed strong associations with CVD risk factors and biomarkers (15).

Despite these advances, the relationship between MetS Score and all-cause mortality remains unclear, especially in older adults. Some studies report linear associations, whereas others suggest paradoxical or J-shaped patterns, possibly reflecting the “obesity paradox” and age-related changes in body composition (16, 17). These uncertainties highlight the importance of evaluating MetS Score in China’s rapidly aging population.

Using data from the Chinese Longitudinal Healthy Longevity Survey (CLHLS), we aimed to assess the association between MetS Score and all-cause mortality in older Chinese adults, with further evaluation of subgroup differences, particularly among those aged ≥80 years, and the potential linearity of this relationship.

## Methods

### Study population

The CLHLS is a nationally representative, prospective cohort of adults aged ≥65 years across 23 provinces of China (18). More detailed information of CLHLS study design can be found at the following website: https://www.icpsr.umich.edu/web/NACDA/studies/38899 (19).

All interviewees were informed and signed the consent form willingly before collecting blood samples and conducting physical examinations. Ethical approval was obtained from the Biomedical Ethics Committee of Peking University (IRB00001052–13074), and all participants provided written informed consent.

**Figure 1:**
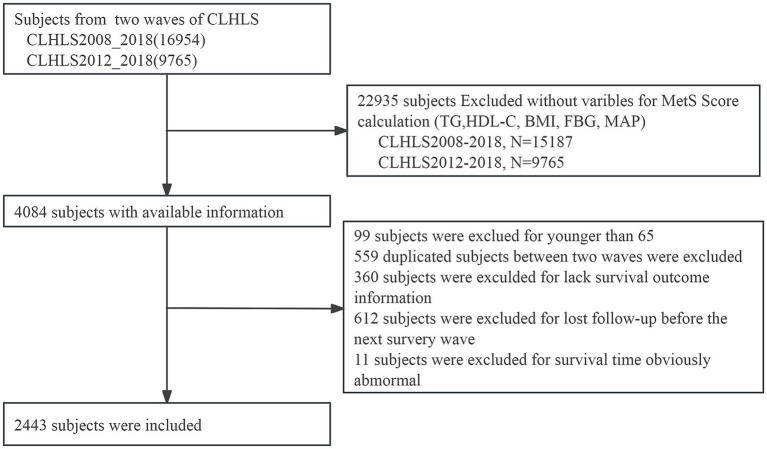
The flowchart of participants enrollment from CLHLS.

For the present analysis, consistent with previous studies using the CLHLS data, older adults were defined as those aged 65 years and above (18). we used three datasets: the CLHLS biomarker dataset, and two follow-up datasets (2008–2018 and 2011–2018). Participants were excluded if they were <65 years of age, lacked biomarker measurements, were duplicated across survey waves, had no survival outcome data, or were lost to follow-up before the subsequent wave. After exclusions, 2,443 participants were eligible and included in the analysis. A detailed flowchart of enrollment and exclusions is presented in Figure 1.

### Data collection

Biological specimens were collected according to standardized protocols, and blood samples were processed and analyzed in certified central laboratories using calibrated equipment (20). Assays included triglycerides (TG), high-density lipoprotein cholesterol (HDL-C), fasting blood glucose (FBG), total cholesterol (TC), and low-density lipoprotein cholesterol (LDL-C).

Physical examinations were conducted by trained physicians from the local Centers for Disease Control (CDC) (19). Measurements comprised systolic blood pressure (SBP), diastolic blood pressure (DBP), height, and weight, from which body mass index (BMI) was calculated as weight in kilograms divided by height in meters squared.

Information on sociodemographic characteristics and lifestyle factors was obtained through structured face-to-face interviews (19). Covariates included age (<80 vs. ≥80 years), sex, ethnicity (Han vs. minority), residence (urban vs. rural), education (illiterate, primary, middle school, high school or above), marital status (married/cohabiting vs. other), living arrangement (alone, institution, or with family), pension status (yes vs. no), smoking (never, former, current), alcohol consumption (never, former, current), and physical activity (never, former, current). The cognitive function of participants at baseline were measured using the Chinese version of the Mini-Mental State Examination (CMMSE) and were catergorized into 2 groups accordding to previous studies (21, 22). Estimated glomerular filtration rate (eGFR) was estimated according to the Chronic Kidney Disease Epidemiology Collaboration (CKD-EPI) equation and catergoried into low eGFR group (< 60 mL/min per 1.73 m^2^) and high eGFR group (≥ 60 mL/min per 1.73 m^2^) (23). The activities of daily living (ADL) disability score, constructed by six daily activities items, including feeding, bathing, dressing, toileting, indoor transferring and continence, were used to measure ADL disability refer to previously studies. The ADL disability score ranged from 0 to 6 points (21, 24). The numbers of chronic diseases of participants were obtained by asking whether they had been diagnosed with hypertension, diabetes mellitus, heart disease, stroke, bronchitis or asthma and cancer.

### Calculation of the MetS Score

A continuous MetS Score was constructed using confirmatory factor analysis (CFA) of five continuous metabolic indicators: TG, HDL-C, FBG, SBP, and BMI (9, 15, 25). Specifically, the skewed indicators including TG, FBG, and BMI were natural log-transformed and HDL-C values was inverted so that higher values of all indicators consistently indicated greater metabolic risk. All five indicators were then standardized and used to estimate the factor loadings in a single-factor CFA model. Finally, following the method of Gurka et al. (25), the final individual MetS Scores equation were derived from the standardized CFA model by back-transforming the estimated coefficients.

### Clinical outcomes

The primary outcome was all-cause mortality, determined from CLHLS follow-up surveys conducted between 2008–2018 and 2011–2018. Vital status and dates of death were obtained during household interviews with close family members or village doctors and, when available, verified against official registration records.

### Statistical analysis

Participants were categorized into quartiles of the MetS Score (Q1-Q4). Baseline characteristics were summarized as mean ± standard deviation (SD) or median (interquartile range, IQR) for continuous variables and as proportions for categorical variables. Differences across quartiles were assessed using analysis of variance (ANOVA) or Kruskal-Wallis tests for continuous variables and *χ*^2^ tests for categorical variables.

The association between MetS Score and all-cause mortality was evaluated using Cox proportional hazards regression, with hazard ratios (HRs) and 95% confidence intervals (CIs) reported. The proportional hazards assumption was tested using Schoenfeld residuals, and no significant violations were observed. Three nested models were specified: Model 1, crude and unadjusted; Model 2, adjusted for age, sex, and ethnicity; and Model 3, further adjusted for residence place, coresidence type, education, marital status, pension status, smoking, drinking, exercising status, survey year, ADL disability score, cognitive function status, eGFR and the number of chronic diseases. Linear trends across quartiles were tested by modeling the median value of each quartile as a continuous variable, as described in previous studies (26).

Prespecified subgroup analyses were conducted by age (<80 vs. ≥80 years), sex, residence place, coresidence type, marital status, pension status, education status, cognitive function status and status of chronic diseases. Restricted cubic spline regression was applied using the MetS Score as a continuous variable, with knots placed at the 5th, 35th, 65th, and 95th percentiles (27). Departure from linearity was tested using likelihood ratio tests.

Missing covariates were addressed using multilevel multiple imputation with chained equations and random effects, accounting for the hierarchical structure of the CLHLS survey data (28). Twenty imputed datasets were created, and final estimates were pooled according to Rubin’s rules (29, 30). To address potential reverse causation, we conducted sensitivity analyses by excluding participants who died within the first year and within the first 2 years of follow-up. All analyses were performed using R software (version 4.4.3), which is open-source and freely available.^1^ A two-sided *p* < 0.05 was considered statistically significant.

## Results

### Development of the MetS Score

Using CFA, a continuous MetS Score was derived from five metabolic indicators (TG, HDL-C, FBG, SBP, and BMI). The fitted formula for participants aged ≥65 years was: MetS Score = −2.095 + 0.003 * SBP + 0.501 * ln(BMI) + 0.673 * ln(FBG) -0.664 * HDL-C + 0.793 * ln(TG).

Factor loadings were highest for TG (0.569), followed by HDL-C (0.394), FBG (0.363), BMI (0.179), and SBP (0.104) (Supplementary Table S1).

### Baseline characteristics of participants

Baseline characteristics of participants across quartiles of MetS Score are summarized in Table 1. Among 2,443 participants, 58.7% were female, and 71.9% were aged ≥80 years. Most were Han (91.2%), rural residents (82.2%), lived with family or in institutions (80.5%), had no pension (92.1%), illiterate (67.50%), had normal cognitive function (CMMSE score ≥18, 76.0%), and had no chronic diseases (69.3%). Nearly 17.7% were current smokers, 17.3% current drinkers, and 16.7% engaged in regular exercise.

**Table 1:** Baseline characteristics of participants from CLHLS according to quartiles of MetS Score.

Characteristics	Total (*n* = 2,443)	Q1 (<−0.44) (*n* = 610)	Q2 (−0.44to −0.07) (*n* = 609)	Q3 (−0.06–0.35) (n = 612)	Q4 (≥0.36) (*n* = 612)	*p*-value
Age (years), median (IQR)	88 (22)	89 (21)	91 (20)	91 (20)	83 (21)	<0.001
Age (years)						<0.001
<80	686 (28.08)	157 (25.74)	142 (23.32)	152 (24.84)	235 (38.40)	
≥80	1757 (71.92)	453 (74.26)	467 (76.68)	460 (75.16)	377 (61.60)	
Sex						<0.001
Male	1,010 (41.34)	290 (47.54)	218 (35.80)	232 (37.91)	270 (44.12)	
Female	1,433 (58.66)	320 (52.46)	391 (64.20)	380 (62.09)	342 (55.88)	
Ethnicity						0.004
Han nationality	2,227 (91.16)	576 (94.43)	558 (91.63)	546 (89.22)	547 (89.38)	
Minority nationality	216 (8.84)	34 (5.57)	51 (8.37)	66 (10.78)	65 (10.62)	
Marital status						<0.001
Married/cohabiting	807 (33.03)	221 (36.23)	170 (27.91)	170 (27.78)	246 (40.20)	
Divorced/never married/separated/widowed	1,636 (66.97)	389 (63.77)	439 (72.09)	442 (72.22)	366 (59.80)	
Residence place						<0.001
Urban	435 (17.81)	93 (15.25)	87 (14.29)	87 (14.21)	168 (27.45)	
Rural	2008 (82.19)	517 (84.75)	522 (85.71)	525 (85.78)	444 (72.55)	
Coresidence type						0.828
Alone	477 (19.53)	127 (20.82)	116 (19.05)	118 (19.28)	116 (18.95)	
Not alone (lived with family members or in an institute)	1966 (80.47)	483 (79.18)	493 (80.95)	494 (80.72)	496 (81.05)	
Education levels						0.009
Illiterate	1,649 (67.50)	399 (65.41)	439 (72.09)	434 (70.92)	377 (61.60)	
Primary school	623 (25.50)	168 (27.54)	133 (21.84)	142 (23.20)	180 (29.41)	
Middle school	112 (4.58)	30 (4.92)	22 (3.61)	24 (3.92)	36 (5.88)	
High school and above	59 (2.42)	13 (2.13)	15 (2.46)	12 (1.96)	19 (3.10)	
Smoking status						0.806
Never	1800 (73.68)	441 (72.30)	464 (76.19)	445 (72.71)	450 (73.53)	
Former	211 (8.64)	57 (9.34)	46 (7.55)	55 (8.99)	53 (8.66)	
Current	432 (17.68)	112 (18.36)	99 (16.26)	112 (18.30)	109 (17.81)	
Drinking status						0.158
Never	1872 (76.63)	453 (74.26)	464 (76.19)	480 (78.43)	475 (77.61)	
Former	149 (6.10)	35 (5.74)	32 (5.25)	37 (6.05)	45 (7.35)	
Current	422 (17.27)	122 (20.00)	113 (18.56)	95 (15.52)	92 (15.03)	
Exercise						<0.001
Never	1919 (78.55)	509 (83.44)	492 (80.79)	478 (78.10)	440 (71.90)	
Former	115 (4.71)	18 (2.95)	30 (4.93)	36 (5.88)	31 (5.07)	
Current	409 (16.74)	83 (13.61)	87 (14.29)	98 (16.01)	141 (23.04)	
BMI						<0.001
Underweight	742 (30.37)	245 (40.16)	197 (32.35)	180 (29.41)	120 (19.61)	
Normal	1,313 (53.75)	327 (53.61)	334 (54.84)	329 (53.76)	323 (52.78)	
Overweight and obesity	388 (15.88)	38 (6.23)	78 (12.81)	103 (16.83)	169 (27.61)	
Pension subsidies						0.001
No	2,251 (92.14)	559 (91.64)	572 (93.92)	577 (94.28)	543 (88.73)	
Yes	192 (7.86)	51 (8.36)	37 (6.08)	35 (5.72)	69 (11.27)	
Survey year						
2008	1,123 (46.0)	86 (14.1)	281 (46.1)	367 (60.0)	389 (63.6)	<0.001
2012	1,320 (54.0)	524 (85.9)	328 (53.9)	245 (40.0)	223 (36.4)	
CMMSE score						
<18	587 (24.0)	130 (21.3)	182 (29.9)	167 (27.3)	108 (17.6)	<0.001
≥ 18	1856 (76.0)	480 (78.7)	427 (70.1)	445 (72.7)	504 (82.4)	
eGFR (ml/min per 1.73 m^2^)						
Low group (<60)	757 (31.0)	144 (23.6)	177 (29.1)	213 (34.8)	223 (36.4)	<0.001
High group (≥ 60)	1,686 (69.0)	466 (76.4)	432 (70.9)	399 (65.2)	389 (63.6)	
Numbers of the self-reported chronic diseases						
0	1,693 (69.3)	436 (71.5)	422 (69.3)	424 (69.3)	411 (67.2)	0.18
1 ~ 2	724 (29.6)	169 (27.7)	181 (29.7)	185 (30.2)	189 (30.9)	
≥ 3	26 (1.1)	5 (0.8)	6 (1.0)	3 (0.5)	12 (2.0)	
The ADL disability score	0.59 (1.44)	0.64 (1.47)	0.65 (1.50)	0.61 (1.42)	0.45 (1.36)	0.062

MetS, metabolic syndrome; Q1–Q4, quartile 1-quartile 4; IQR, interquartile range; BMI, body mass index. ADL, activities of daily living; CMMSE, the Chinese version of the Mini Mental State Examination; eGFR, Estimated glomerular filtration rate.

Quartile 1: MetS<−0.44, Quartile 2: −0.44 < MetS<−0.07, Quartile 3: −0.06 < MetS<0.35, Quartile 4: MetS≥0.36.

Median (IQR) MetS Scores were −0.70 (0.37) for Q1, −0.24 (0.18) for Q2, 0.14 (0.20) for Q3, and 0.75 (0.63) for Q4. Participants in Q4 were younger, more likely to be female, urban residents, married or cohabiting, overweight/obese, and more likely to receive pensions, engage in physical activity, with lower eGFR and normal cognitive function (CMMSE score ≥18).

### Association between the MetS Score and all-cause mortality

**Figure 2:**
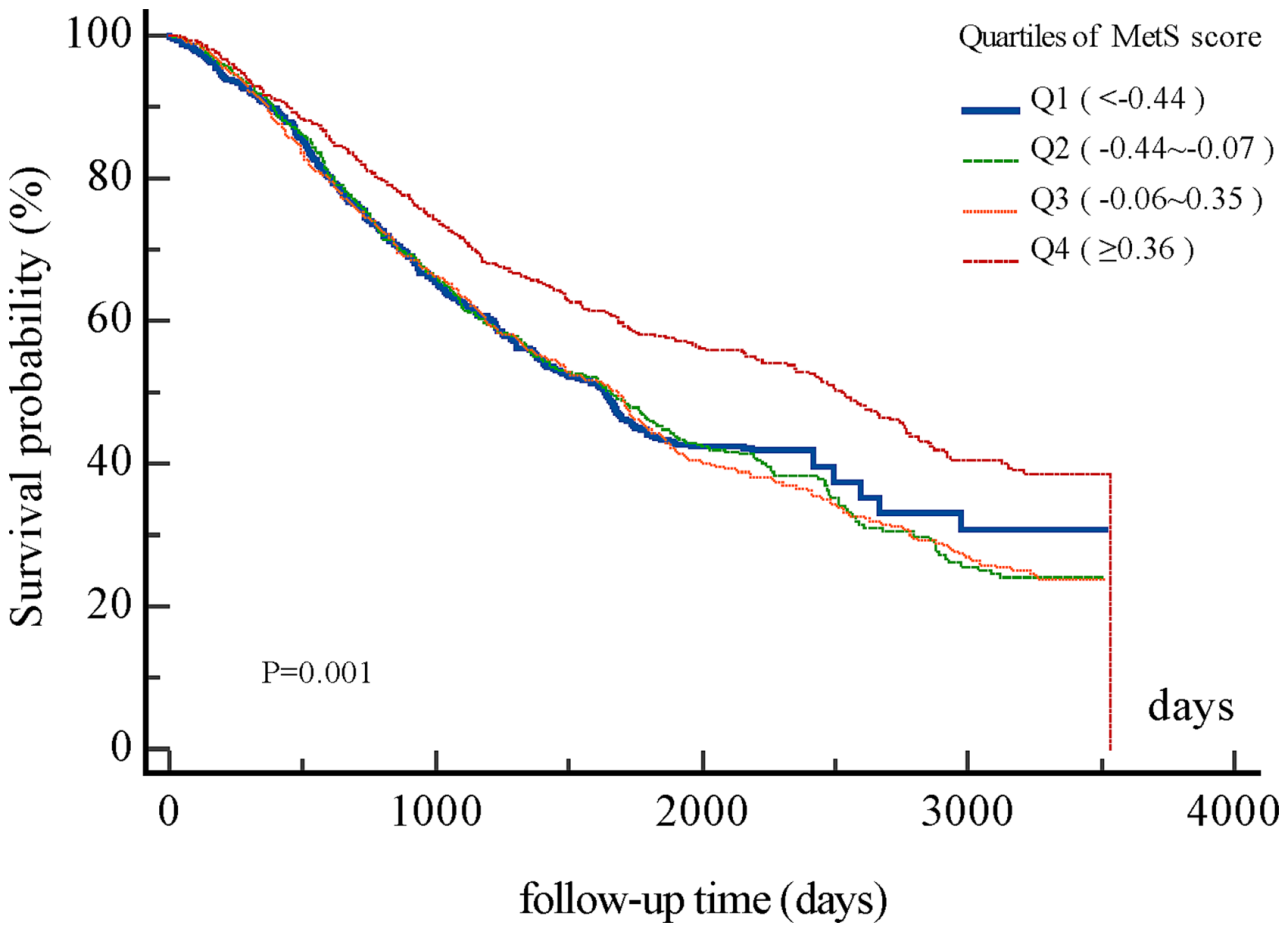
The Kaplan–Meier survival analysis curve for all-cause mortality according to quartiles of MetS Score.

Kaplan–Meier survival curves illustrating mortality across quartiles of MetS Score are shown in Figure 2. During 10,356.6 person-years of follow-up (median 3.8 years), 1,412 deaths occurred, corresponding to a crude mortality rate of 136.3 per 1,000 person-years. Mortality rates by quartile were 145.2, 148.8, 153.8, and 103.1 per 1,000 person-years for Q1-Q4, respectively. Kaplan–Meier curves demonstrated significant differences across quartiles (*P*_for-log-rank_ = 0.001).

Table 2 presents the results of Cox proportional hazards regression models examining the association between MetS Score and all-cause mortality. Each one-unit increase in MetS Score was associated with a 19.6% reduction in mortality in the crude model (HR = 0.804, 95% CI: 0.743–0.869, *p* < 0.001). After adjustment for age, sex, and ethnicity, the association remained significant though attenuated (HR = 0.864, 95% CI: 0.796–0.938, *p* < 0.001). In the fully adjusted model, the protective association persisted (HR = 0.861, 95% CI: 0.787–0.942, *p* = 0.001).

**Table 2:** The hazard ratios for the association between MetS Score and all-cause mortality (*N* = 2,443).

Models	HR (95%CI)	*p*-value	HR (95%CI)				*P* for trend
	As continuous variable		Q1	Q2	Q3	Q4	
Model 1	0.804 (0.743–0.869)	<0.001	Ref	1.023 (0.883–1.186)	1.056 (0.913–1.222)	0.707 (0.605–0.828)	<0.001
Model 2	0.864 (0.796–0.938)	<0.001	Ref	0.968 (0.835–1.123)	1.026 (0.886–1.187)	0.804 (0.687–0.942)	0.012
Model 3	0.861 (0.787–0.942)	0.001	Ref	0.939 (0.804–1.096)	0.979 (0.835–1.149)	0.792 (0.669–0.938)	0.009

MetS, metabolic syndrome; HR, hazard ratios; 95% CI, 95% confidence interval; ref, reference.

Model 1: crude model, no co-variables was adjusted; Model 2: adjusted for age, sex and ethnicity; Model 3: further adjusted for residence area, coresidence type, educational levels, current marital status, pension subsidies, smoking status, drinking status, exercising status, survey year, ADL disability score, cognitive function, eGFR and the numbers of chronic diseases.

**Figure 3:**
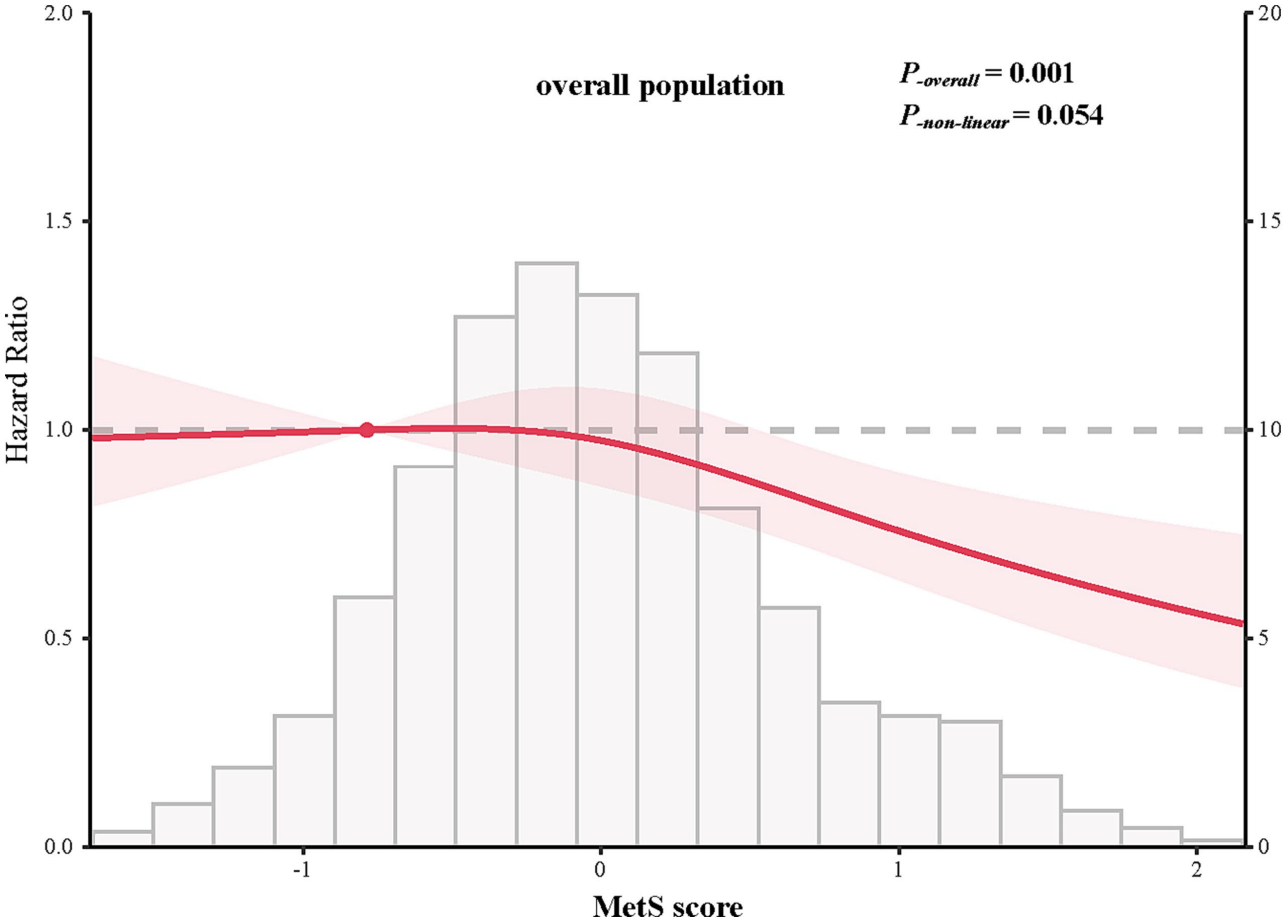
Restricted cubic spline model of the association between MetS Score and all-cause mortality in overall population.

When analyzed by quartiles, the highest quartile (Q4) demonstrated a consistent protective effect compared with Q1. The hazard ratios for Q4 were 0.707 (95% CI: 0.605–0.828, *p* < 0.001) in Model 1, 0.804 (95% CI: 0.687–0.942, *p* = 0.012) in Model 2, 0.792 (95% CI: 0.669–0.938, *p* = 0.009) in Model 3 (Table 2). Further sensitivity analyses in 2219 older adults demonstrated consistent results, confirming the stability of the observed associations (Supplementary Table S2). Kaplan–Meier survival curves (Figure 2) further illustrated the lower cumulative mortality risk in Q4 compared with the other quartiles. Restricted cubic spline analysis (Figure 3) confirmed a linear inverse dose–response association between MetS Score and mortality (*P*-overall = 0.001, *P*-nonlinear = 0.054).

### Subgroup analysis

Tables 3, 4 summarize the subgroup analyses of the association between MetS Score and all-cause mortality. As a continuous variable, the MetS Score was significantly protective in several subgroups: those aged ≥80 years (HR = 0.847, 95% CI: 0.769–0.933, *p* < 0.001), women (HR = 0.839, 95% CI: 0.745–0.944, *p* = 0.003), unmarried individuals (divorced/never married/separated/widowed) (HR = 0.835, 95% CI: 0.753–0.927, *p* < 0.001), rural residents (HR = 0.870, 95% CI: 0.784–0.966, *p* = 0.009), those lived with family members or in institutions (HR = 0.847, 95% CI: 0.767–0.935, *p* < 0.001), those without pension subsidies(HR = 0.848, 95% CI: 0.772–0.931, *p* < 0.001), those with CMMSE score <18 (HR = 0.709, 95% CI: 0.592–0.848, *p* < 0.001), those with eGFR ≥60 mL/min per 1.73 m^2^ (HR = 0.848, 95% CI: 0.747–0.962, *p* = 0.010) and those without self-reported chronic diseases (HR = 0.784, 95% CI: 0.697–0.881, *p* < 0.001). Protective effects were evident in both illiterate participants (HR = 0.879, 95% CI: 0.791–0.978, *p* = 0.018) and those who received formal education (HR = 0.813, 95% CI: 0.675–0.978, *p* = 0.028).

**Table 3:** Subgroup analysis of the association between MetS Score (as continuous variable) and the risk of all-cause mortality.

Characteristics	Event/no.	As continuous variable	*p*-value
		HR (95%CI)	
Age (years)			
<80	131/686	0.836 (0.638–1.096)	0.195
≥80	1281/1757	0.847 (0.769–0.933)	<0.001
Sex			
Male	499/1010	0.907 (0.783–1.051)	0.194
Female	913/1433	0.839 (0.745–0.944)	0.003
Marital status			
Married/cohabiting	259/807	0.958 (0.789–1.161)	0.659
Divorced/never married/separated/widowed	1153/1636	0.835 (0.753–0.927)	<0.001
Residence place			
Rural	1172/2008	0.870 (0.784–0.966)	0.009
Urban	240/435	0.820 (0.672–1.001)	0.052
Coresidence type			
Alone	260/477	0.892 (0.708–1.122)	0.328
Not alone	1152/1966	0.847 (0.767–0.935)	<0.001
Pension subsidies			
No	1341/2251	0.848 (0.772–0.931)	<0.001
Yes	71/192	1.175 (0.831–1.663)	0.362
Education levels			
Illiterate	1105/1649	0.879 (0.791–0.978)	0.018
Formal education	307/794	0.813 (0.675–0.978)	0.028
CMMSE score			
<18	516/587	0.709 (0.592–0.848)	<0.001
≥ 18	896/1856	0.927 (0.832–1.032)	0.166
eGFR (mL/min per 1.73 m^2^)			
low group (<60)	544/757	0.880 (0.770–1.005)	0.060
high group (≥ 60)	868/1686	0.848 (0.747–0.962)	0.010
The self-reported chronic diseases			
No	985/1693	0.784 (0.697–0.881)	<0.001
Yes	427/750	0.984 (0.850–1.139)	0.830

MetS, metabolic syndrome; HR, hazard ratios; 95% CI, 95% confidence interval; BMI, body mass index.

Model was adjusted for age, sex, ethnicity, residence area, coresidence type, educational levels, current marital status, pension subsidies, smoking status, drinking status, exercising status, survey year, ADL disability score, cognitive function, eGFR and the numbers of chronic diseases.

**Table 4:** Subgroup analysis of the association between quartiles of MetS Score and the risk of all-cause mortality.

Characteristics	Event/No.	Q1	HR (95%CI)			*P* for trend
			Q2	Q3	Q4	
Age (years)						
<80	131/686	Ref	1.348 (0.761–2.388)	1.051 (0.578–1.913)	1.033 (0.551–1.934)	0.703
≥80	1281/1757	Ref	0.943 (0.801–1.111)	0.919 (0.774–1.089)	0.785 (0.660–0.935)	0.005
Sex						
Male	499/1010	Ref	1.080 (0.834–1.398)	1.114 (0.848–1.462)	0.939 (0.702–1.257)	0.605
Female	913/1433	Ref	0.898 (0.742–1.087)	0.931 (0.765–1.132)	0.758 (0.617–0.931)	0.012
Marital status						
Married/cohabiting	259/807	Ref	1.064 (0.737–1.537)	1.168 (0.796–1.713)	1.046 (0.688–1.591)	0.813
Divorced/never married/separated/widowed	1153/1636	Ref	0.954 (0.804–1.133)	0.914 (0.765–1.092)	0.798 (0.665–0.958)	0.011
Residence place						
Rural	1172/2008	Ref	0.922 (0.776–1.096)	0.837 (0.700–1.000)	0.817 (0.681–0.980)	0.020
Urban	240/435	Ref	1.096 (0.737–1.630)	1.436 (0.953–2.162)	0.559 (0.336–0.930)	0.055
Coresidence type						
Alone	260/477		0.732 (0.494–1.084)	0.828 (0.557–1.23)	0.823 (0.544–1.245)	0.627
Not alone (lived with family members or in an institute)	1152/1966		1.030 (0.868–1.222)	1.029 (0.862–1.228)	0.791 (0.655–0.956)	0.008
Pension subsidies						
No	1341/2251	Ref	0.978 (0.833–1.148)	0.983 (0.833–1.160)	0.793 (0.667–0.944)	0.007
Yes	71/192	Ref	1.866 (0.883–3.941)	2.905 (1.292–6.534)	1.710 (0.714–4.096)	0.178
Education levels						
Illiterate	1105/1649	Ref	1.002 (0.839–1.195)	0.993 (0.825–1.195)	0.883 (0.731–1.066)	0.157
Formal education	307/794	Ref	1.027 (0.736–1.432)	1.005 (0.715–1.412)	0.749 (0.509–1.103)	0.130
CMMSE score						
<18	516/587	Ref	0.740 (0.573–0.955)	0.731 (0.561–0.952)	0.633 (0.483–0.829)	0.002
≥ 18	896/1856	Ref	0.997 (0.82–1.212)	1.110 (0.906–1.359)	0.920 (0.741–1.144)	0.518
eGFR (mL/min per 1.73 m^2^)						
Low group (<60)	544/757	Ref	0.832 (0.647–1.068)	1.101 (0.858–1.412)	0.700 (0.536–0.915)	0.032
High group (≥ 60)	868/1686	Ref	0.957 (0.787–1.164)	0.969 (0.787–1.194)	0.873 (0.701–1.088)	0.245
The self-reported chronic diseases						
No	985/1693	Ref	0.889 (0.738–1.072)	0.911 (0.751–1.105)	0.699 (0.569–0.859)	<0.001
Yes	427/750	Ref	1.118 (0.84–1.488)	1.137 (0.846–1.529)	1.029 (0.756–1.400)	0.947

MetS, metabolic syndrome; HR, hazard ratios; 95% CI, 95% confidence interval; BMI, body mass index, ref, reference.

Model was adjusted for age, sex, ethnicity, residence area, coresidence type, educational levels, current marital status, pension subsidies, smoking status, drinking status, exercising status survey year, ADL disability score, cognitive function, eGFR and the numbers of chronic diseases.

When analyzed categorically, the protective effect remained consistent across most subgroups (Table 4). Compared with Q1, Q4 was significantly associated with lower mortality risk in participants aged ≥80 years (HR = 0.785, 95% CI: 0.660–0.935, *P_-for-trend_* = 0.005), women (HR = 0.758, 95% CI: 0.617–0.931, *P_-for-trend_* = 0.012), unmarried individuals(HR = 0.798, 95% CI: 0.665–0.958, *P_-for-trend_* = 0.011), rural residents (HR = 0.817, 95% CI: 0.618–0.980, *P_-for-trend_* = 0.020), those living with family members or in institutions(HR = 0.791, 95% CI:0.655–0.956, *P_-for-trend_* = 0.008), those without pension subsidies(HR = 0.793, 95% CI: 0.667–0.944, *P_-for-trend_* = 0.007) and those without chronic diseases (HR = 0.699, 95% CI: 0.569–0.859, *P_-for-trend_* <0.001).

In addition, restricted cubic spline analysis (Supplementary Figure S1) also confirmed the association between MetS Score and mortality among different subgroups.

### Sensitivity and validation analyses

In sensitivity analyses, the inverse association between MetS Score and all-cause mortality remained consistent after excluding deaths within the first year and within the first 2 years of follow-up (Supplementary Tables S2, S3). Bootstrap internal validation showed stable CFA factor loadings and no substantial overfitting, with an optimism-corrected Harrell’s C-index similar to the apparent C-index (Supplementary Table S4). Incremental discrimination analyses showed that adding the MetS Score to the fully adjusted model produced a modest increase in Harrell’s C-index from 0.762 to 0.766 (Supplementary Table S5).

### The associations between MetS Score and all-cause mortality in older population aged ≥80 years

Table 5 presents the results of Cox models restricted to the 1,757 participants aged ≥80 years. In this subgroup, each one-unit increase in MetS Score was associated with a 15.3% lower mortality risk in the fully adjusted model (HR = 0.847, 95% CI: 0.769–0.933, *p* < 0.001). Compared with Q1, those in Q4 consistently had lower mortality risk across all models: HR = 0.794 (95% CI: 0.677–0.932, *P*_-for-trend_ = 0.003) in Model 1, HR = 0.794 (95% CI: 0.676–0.932, *P*_-for-trend_ = 0.003) in Model 2, and HR = 0.785 (95% CI: 0.660–0.935, *P*_-for-trend_ = 0.005) in Model 3.

**Table 5:** Associations between MetS Score and the risk of all-cause mortality in adults aged 80 and above (*N* = 1757).

Models	As continuous variable	*p*-value	Q1	Q2	Q3	Q4	*P* for trend
	HR (95%CI)		HR (95%CI)				
Model 1	0.841 (0.770–0.918)	<0.001	Ref	0.962 (0.823–1.124)	0.925 (0.792–1.081)	0.794 (0.677–0.932)	0.003
Model 2	0.840 (0.769–0.917)	<0.001	Ref	0.956 (0.817–1.118)	0.920 (0.787–1.075)	0.794 (0.676–0.932)	0.003
Model 3	0.847 (0.769–0.933)	<0.001	Ref	0.943 (0.801–1.111)	0.919 (0.774–1.089)	0.785 (0.660–0.935)	0.005

MetS, metabolic syndrome; HR, hazard ratios; 95% CI, 95% confidence interval; ref, reference.

Model 1: crude model; Model 2:adjusted for sex and ethnicity; Model 3:further adjusted for residence area, coresidence type, educational levels, current marital status, pension subsidies, smoking status, drinking status, exercising status, survey year, ADL disability score, cognitive function, eGFR and the numbers of chronic diseases.

In addition, restricted cubic spline analysis (Supplementary Figure S1B) confirmed a linear inverse association between MetS Score and mortality among the oldest-old (*P*-overall = 0.002; *P*-nonlinear = 0.058).

## Discussion

In this large, nationally representative cohort of older Chinese adults, we found that a higher MetS Score was inversely associated with all-cause mortality. This protective association was particularly evident among the oldest-old (≥80 years), women, unmarried participants and rural residents, and persisted after multivariable adjustment. When analyzed by quartiles, only participants in the highest quartile (Q4) consistently demonstrated lower mortality compared with Q1. Restricted cubic spline analyses confirmed a linear inverse relationship in the overall population and in the oldest-old subgroup.

Our findings provide new insights into the relationship between MetS severity and mortality in older adults. First, we observed that higher continuous MetS Scores were associated with lower all-cause mortality. This is in contrast to many studies conducted in middle-aged populations, where both traditional MetS definitions and continuous severity scores have been positively associated with incident CVD and mortality (12, 31). For example, analyses from the U.S. NHANES cohort and the Iranian Lipid and Glucose Study demonstrated that each unit increase in MetS severity score predicted higher risk of death and cardiovascular events, often with linear or J-shaped patterns (12, 32). Only participants in the highest quartile (Q4) showed a consistent survival benefit. In younger populations, mortality generally rises across the full gradient of MetS severity (33), whereas in our older sample the protective effect was limited to those with the greatest metabolic load. This pattern may reflect the “obesity paradox,” where greater adiposity and metabolic reserves become advantageous in late life (34). This may be because elevated TG and BMI reflect better nutritional status and energy reserves, protecting against malnutrition, sarcopenia, and frailty—key non-cardiovascular diseases mortality drivers in older adults (34–36).

Subgroup analyses showed stronger inverse associations among the oldest-old (≥80 years), women, and rural residents. Prior studies suggest that survival benefits of higher BMI and certain metabolic traits are more evident in women and the very old (16, 37), while urban–rural differences may reflect disparities in healthcare access and socioeconomic conditions. Our results extend recent work in China that developed an age-, sex-, and ethnicity-specific MetS Score and showed strong associations with CVD-related biomarkers (15). By linking this score to mortality outcomes, we provide novel evidence that the prognostic meaning of MetS severity differs by age, with paradoxical survival benefits emerging in the oldest-old.

Several mechanisms may underlie the paradoxical finding that higher MetS Scores were associated with lower mortality among older adults. One explanation is the well-described “obesity paradox,” whereby overweight or mildly obese individuals, especially in late life, demonstrate better survival than those with normal weight. Meta-analyses of patients with heart failure and other chronic conditions consistently show that higher BMI confers a survival advantage in older populations (38). A second explanation relates to nutritional and body-composition reserves: higher MetS Scores may partly capture greater fat and lean mass, which can provide essential energy and immunometabolic reserves during illness or stress. Epidemiologic studies indicate that sarcopenia is a strong predictor of mortality in older adults (39) and that inflammation-related muscle loss contributes to frailty and adverse outcomes (40). In this context, individuals with higher metabolic load but preserved muscle may have enhanced resilience compared with leaner counterparts. A third consideration is survivorship bias: those who reach advanced ages despite long-standing metabolic abnormalities may constitute a biologically robust subgroup with protective genetic, immunologic, or behavioral factors. Such selection effects have been widely discussed in epidemiologic methodology (41). Collectively, these mechanisms suggest that while high MetS severity increases cardiometabolic risk in younger populations, its prognostic meaning may shift in late life, where greater metabolic and nutritional reserves, combined with selective survival, could paradoxically indicate improved longevity.

Our findings have direct implications for geriatric risk assessment and management. In younger and middle-aged populations, continuous MetS Scores are valuable tools for identifying individuals at elevated cardiometabolic risk. However, in the oldest-old, their interpretation requires caution, as a higher score may not uniformly indicate vulnerability and may in some contexts reflect protective metabolic or nutritional reserves. These results underscore the importance of moving away from a “one-size-fits-all” threshold and adopting age-specific approaches to metabolic risk stratification. Clinicians should consider the broader physiological and social context of older adults when applying MetS-based tools, and policymakers should incorporate these nuances into guidelines for chronic disease prevention and healthy aging.

This study has several notable strengths, including the use of a large, nationally representative cohort of older Chinese adults, long-term follow-up with validated mortality outcomes, and application of a CFA-derived MetS Score that reflects metabolic severity on a continuous scale. Nonetheless, limitations should be acknowledged. First, residual confounding cannot be fully excluded despite extensive adjustment for demographic, socioeconomic, and lifestyle factors. Second, BMI was used as the adiposity measure; although practical, it does not distinguish between fat and lean mass, and future studies should incorporate waist circumference or imaging-based assessments of visceral fat. Third, the MetS Score was originally designed for cardiovascular risk prediction, and its applicability to all-cause mortality in adults aged ≥80 years may be limited, given that mortality in this population is driven by non-cardiovascular factors (e.g., infections, frailty, sarcopenia). Future studies are needed to validate the incremental predictive value of the MetS Score for all-cause mortality in the oldest-old. Fourth, the MetS Score was calculated using within-sample standardization of the five metabolic indicators rather than an external reference population, which may limit comparability with other studies using different reference standards or scoring methods. Fifth, selective survival bias and limited sample representativeness (2,443 participants, <25% of the original cohort) may restrict generalizability. Our findings are more likely to apply to relatively healthy, urban-dwelling older adults with higher socioeconomic status, rather than the general older population. Finally, cause-specific mortality could not be assessed, limiting our ability to determine whether the protective association was driven primarily by cardiovascular or non-cardiovascular deaths.

## Conclusion

In conclusion, higher MetS Scores were paradoxically associated with lower all-cause mortality among older Chinese adults, particularly in the oldest-old subgroup. These findings highlight that the prognostic meaning of MetS severity differs across the life course and emphasize the need for age-tailored strategies in metabolic risk assessment and prevention.

## Data Availability

Publicly available datasets were analyzed in this study. This data can be found at: https://www.icpsr.umich.edu/web/NACDA/studies/38899.

## Footnotes

1
https://www.r-project.org/

## Supplementary material

SUPPLEMENTARY FIGURE S1 **(A–T)** Restricted cubic spline models of the association between MetS Score and all-cause mortality in different predefined subgroups.
