# Supplementary figures and images for: Metabolic syndrome severity and all-cause mortality in the CLHLS biomarker subsample of older Chinese adults
Source: Front Public Health. 2026 May 29;14:1832339. doi: 10.3389/fpubh.2026.1832339 (PMC13259693; doi:10.3389/fpubh.2026.1832339)

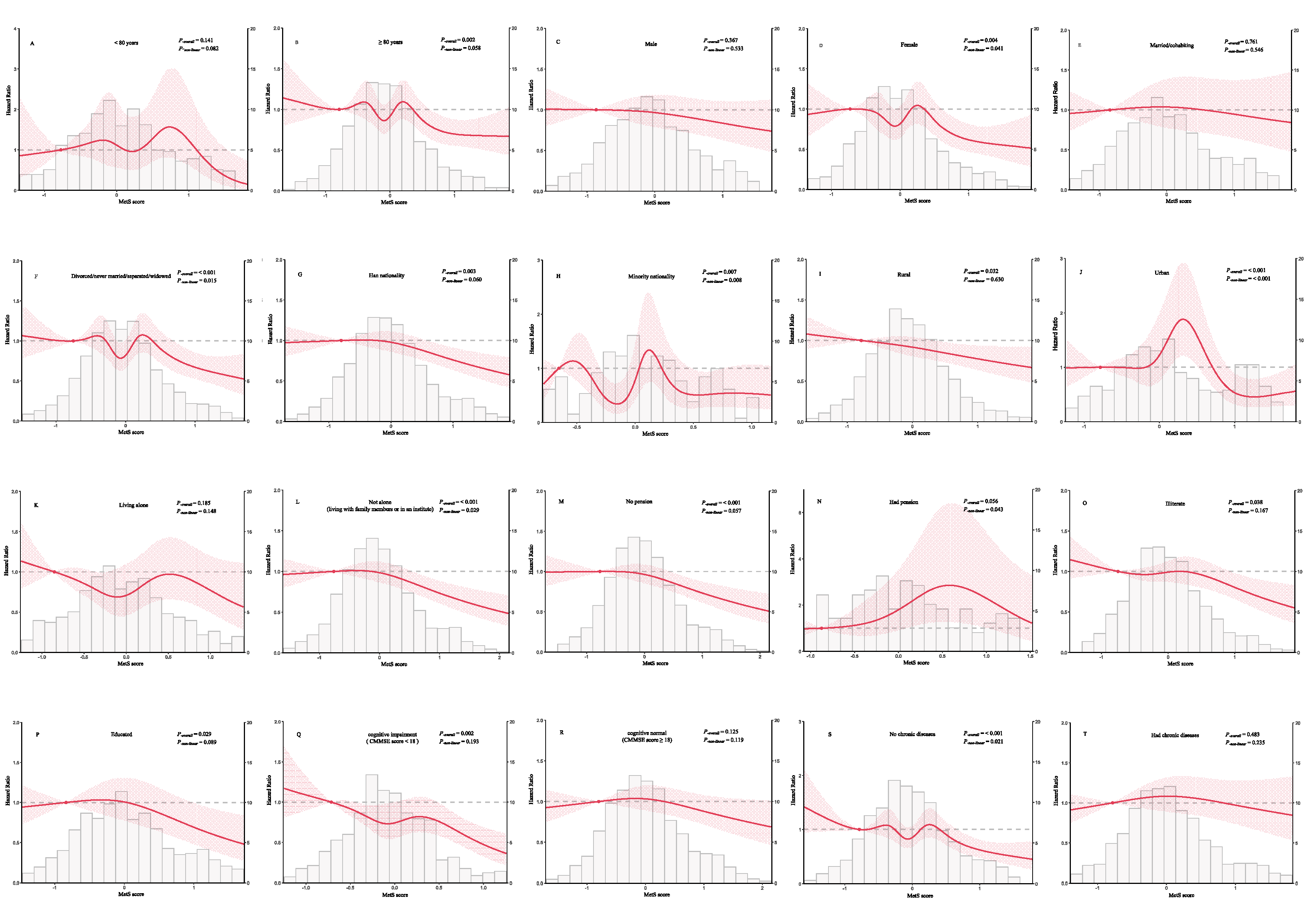

Supplement: SUPPLEMENTARY FIGURE S1 — (A–T) Restricted cubic spline models of the association between MetS Score and all-cause mortality in different predefined subgroups. [file Image_1.tiff]
