# Supplementary material for: Metabolic syndrome severity and all-cause mortality in the CLHLS biomarker subsample of older Chinese adults
Source: Front Public Health. 2026 May 29;14:1832339. doi: 10.3389/fpubh.2026.1832339 (PMC13259693; doi:10.3389/fpubh.2026.1832339)
Supplement: Supplementary file 2 [file Table_1.docx]

**Supplementary Table S1. Model fitting results for Confirmatory Factor Analysis**

|  | **Model Fit Indices** | **Cut-off for good fit** | **Results** |
| --- | --- | --- | --- |
| CMIN | 18.803 | - | - |
| SRMR | 0.020 | <0.08 | Good |
| RMSEA | 0.008 | <0.08 | Good |
| GFI | 0.997 | ≥0.95 | Good |
| CFI | 0.956 | ≥0.90 | Good |
| NFI | 0.942 | ≥0.95 | Acceptable |

CMIN, chi-square minimum discrepancy statistic;

SRMR:Standardized Root Mean Square Residual;

GFI: Goodness of Fit Index;

CFI: Comparative Fit Index;

NFI: Bentler-Bonett Normed Fit,

RMSEA:Root Mean Square Error of Approximation.

**Supplementary Table S2. Results of sensitivity analysis by excluding participants died during the first year of follow-up (N = 2219)**

|  | **as continuous score** | ***P-value*** | **Q1** | **Q2** | **Q3** | **Q4** | ***P-for-trend*** |
| --- | --- | --- | --- | --- | --- | --- | --- |
|  | **HR (95% CI)** |  | **HR (95% CI)** | | | |  |
| model 1 | 0.783 (0.719-0.853) | <0.001 | ref | 1.049 (0.893-1.232) | 1.057 (0.901-1.239) | 0.685 (0.577-0.815) | <0.001 |
| model 2 | 0.837 (0.765-0.915) | <0.001 | ref | 0.997 (0.848-1.172) | 1.017 (0.867-1.193) | 0.779 (0.655-0.927) | 0.005 |
| model 3 | 0.809 (0.732-0.894) | <0.001 | ref | 0.946 (0.799-1.121) | 0.924 (0.775-1.101) | 0.738 (0.612-0.890) | <0.001 |

HR: Hazard ratio; 95% CI: 95% Confidence Interval.

**Supplementary Table S3. Sensitivity analysis excluding participants who died within the first 2 years of follow-up**

| **Model** | **Continuous MetS Score HR (95% CI)** | **P value** | **Q1** | **Q2 HR (95% CI)** | **Q3 HR (95% CI)** | **Q4 HR (95% CI)** | **P for trend** |
| --- | --- | --- | --- | --- | --- | --- | --- |
| Model 1 | 0.785 (0.716–0.861) | <0.001 | ref | 1.041 (0.864–1.255) | 1.018 (0.843–1.229) | 0.681 (0.557–0.833) | <0.001 |
| Model 2 | 0.843 (0.766–0.928) | <0.001 | ref | 0.992 (0.821–1.198) | 0.981 (0.811–1.188) | 0.761 (0.621–0.932) | 0.006 |
| Model 3 | 0.824 (0.741–0.916) | <0.001 | ref | 0.951 (0.778–1.163) | 0.936 (0.760–1.154) | 0.752 (0.603–0.938) | 0.008 |

Model 1 was unadjusted. Model 2 was adjusted for age, sex, and ethnicity. Model 3 was further adjusted for residence, living arrangement, education, marital status, pension status, smoking, drinking, physical activity, survey year, ADL disability score, cognitive function, eGFR, and number of chronic diseases. HR, hazard ratio; CI, confidence interval; ref, reference.

**Supplementary Table S4. Internal validation of the MetS Score and Cox model**

**Supplementary Table S4A. Bootstrap stability of CFA factor loadings**

| **MetS component** | **Original factor loading** | **Bootstrap mean** | **Bootstrap 95% percentile interval** |
| --- | --- | --- | --- |
| ln(TG) | 0.569 | 0.566 | 0.519–0.614 |
| Inverted HDL-C | 0.394 | 0.391 | 0.346–0.438 |
| ln(FBG) | 0.363 | 0.359 | 0.314–0.407 |
| ln(BMI) | 0.179 | 0.181 | 0.135–0.226 |
| SBP | 0.104 | 0.107 | 0.061–0.153 |

Note: Bootstrap validation was performed with 500 resamples. The rank order and magnitude of factor loadings were materially consistent with the original CFA model, suggesting acceptable internal stability of the cohort-specific MetS Score.

**Supplementary Table S4B. Bootstrap validation of the association with all-cause mortality**

| **Analysis** | **Original estimate** | **Bootstrap median** | **Bootstrap 95% percentile interval** |
| --- | --- | --- | --- |
| Per 1-unit increase in MetS Score | 0.861 | 0.858 | 0.782–0.946 |
| Q4 vs Q1 | 0.792 | 0.789 | 0.657–0.952 |
| Apparent Harrell’s C-index | 0.766 | — | — |
| Optimism-corrected C-index | 0.761 | — | 0.747–0.775 |
| Estimated optimism | 0.005 | — | — |

Note: Values are hazard ratios unless otherwise specified. Bootstrap internal validation was based on 500 resamples. The optimism-corrected C-index was similar to the apparent C-index, suggesting no substantial overfitting. Abbreviations: BMI, body mass index; CFA, confirmatory factor analysis; FBG, fasting blood glucose; HDL-C, high-density lipoprotein cholesterol; MetS, metabolic syndrome; SBP, systolic blood pressure; TG, triglycerides.

**Supplementary Table S5. Incremental predictive performance of the MetS Score**

| **Model** | **Variables included** | **Harrell's C-index** | **95% CI** | **Δ C-index** |
| --- | --- | --- | --- | --- |
| Model A | Age + sex + ethnicity | 0.731 | 0.717–0.745 | Reference |
| Model B | Model A + BMI | 0.734 | 0.720–0.748 | +0.003 |
| Model C | Model A + ADL score + CMMSE + eGFR + chronic disease number | 0.758 | 0.744–0.772 | +0.027 |
| Model D | Fully adjusted model without MetS Score | 0.762 | 0.748–0.776 | Reference |
| Model E | Fully adjusted model + MetS Score | 0.766 | 0.752–0.780 | +0.004 |

The fully adjusted model included age, sex, ethnicity, residence, living arrangement, education, marital status, pension status, smoking, drinking, physical activity, survey year, ADL disability score, cognitive function, eGFR, and number of chronic diseases. Δ C-index was calculated relative to the indicated reference model. ADL, activities of daily living; CMMSE, Chinese Mini-Mental State Examination; eGFR, estimated glomerular filtration rate; CI, confidence interval.
